# Magnetic resonance safety assessment of a new trend: magnetic eyelashes

**DOI:** 10.1002/acm2.12952

**Published:** 2020-06-20

**Authors:** Cihat Eldeniz, Trevor Andrews, Uday Krishnamurthy, Lamyaa Aljaafari, Glenn Foster, Tammie L. S. Benzinger, Hongyu An, Pamela K. Woodard

**Affiliations:** ^1^ Mallinckrodt Institute of Radiology Washington University School of Medicine St. Louis MO USA; ^2^ Siemens Healthineers St. Louis MO USA; ^3^ Department of Medical Imaging and Radiation Therapeutics Saint Louis University St. Louis MO USA

## 1 INTRODUCTION

One type of cosmetic that has gained recent popularity is the magnetic false eyelash. Some of these magnetic eyelashes are placed onto magnetic eyeliner applied to eye lids, while others come in pairs and clamp around the natural eyelashes. This presents potential safety hazards in the magnetic resonance imaging (MRI) environment. This is because patients may not deem it necessary to disclose this type of cosmetic, the standard MR screening forms often fail to capture these accessories, and the eyelashes may not be readily noticed by the technologists.

MRI artifacts caused by magnetic eyelashes were reported previously.^1^ However, in this study, we used industry standard testing methods specifically to examine the projectile risk. We quantified the deflection force experienced by magnetic eyelashes from various manufacturers. All tested products produced large deflections, indicative of significant magnetic forces. The translational forces were so strong that other effects (such as rotational forces, heating, and artifacts) could only be tested in a manner that is inconsistent with how a patient would present in a usual clinical setting.

## 2 MATERIALS AND METHODS

All deflection tests were performed on a 3‐Tesla MR scanner (MR component, Biograph mMR, Siemens Healthcare, Erlangen, Germany) following the guidelines listed in ASTM F2052‐15.^2^ Because it was presumed that the magnetic eyelashes would not remain in place as the subject moved into the isocenter of the bore, no heating or torque testing was performed.

**Fig. 1:**
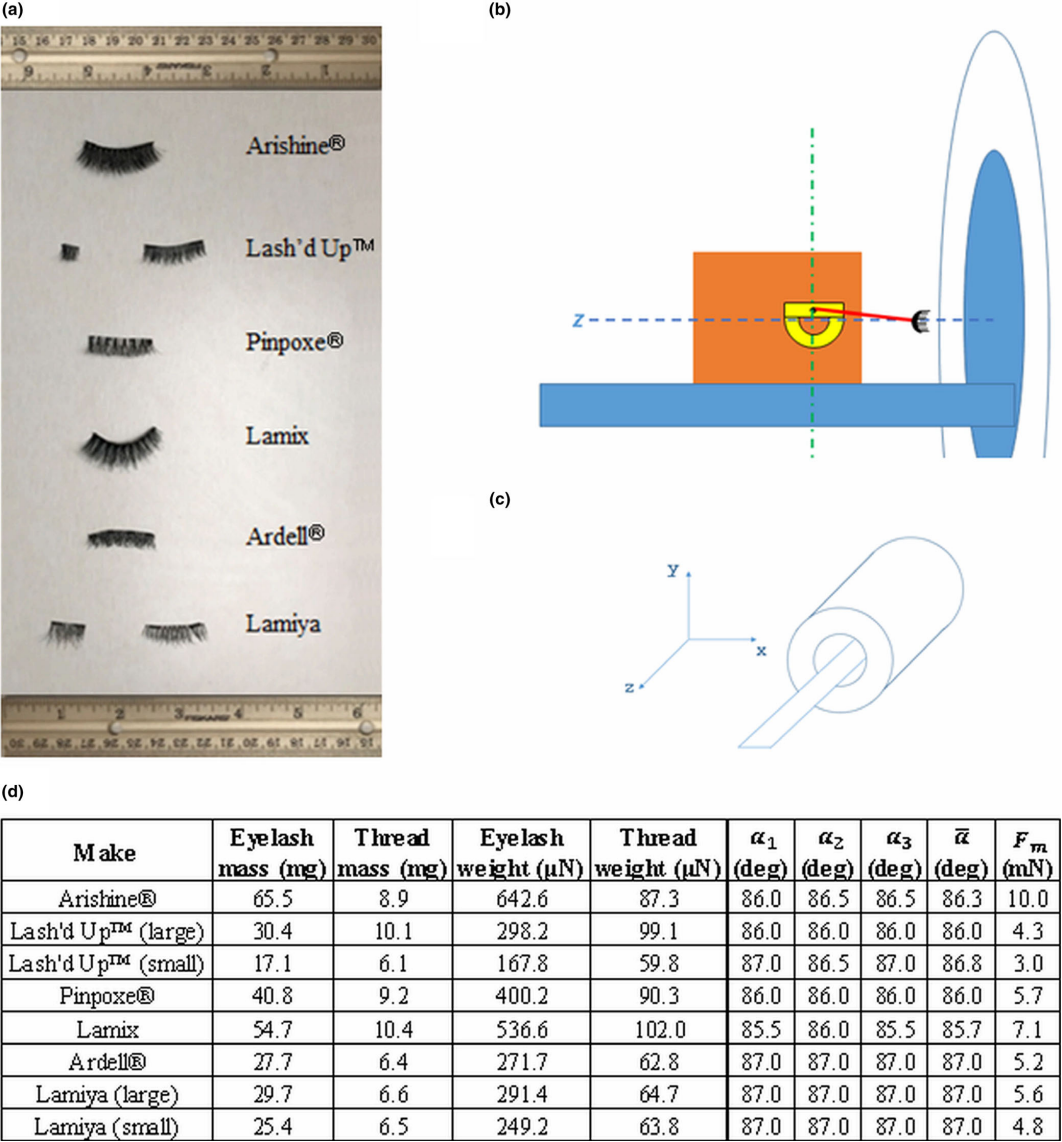
(a) Magnetic eyelash brands that were tested. The Lash'd Up™ and Lamiya sets contain two different sizes of eyelashes for clasping; therefore, both sizes were tested. (b) The setup for magnetically induced deflection force measurement. The foam padding (orange) provides support for the protractor (yellow) to keep it straight. The dashed blue line indicates the z‐axis of the scanner. The string (red) is tied to the protractor and can rotate freely while holding the eyelash (black). The angle between the vertical green dash‐dot line and the string yields the deflection angle. (c) Diagram showing scanner coordinates. (d) Magnetically induced deflection force measurements for each make of magnetic eyelash. $\alpha_{1}$, $\alpha_{2}$, and $\alpha_{3}$are the deflection angles obtained by repeating the measurement three times. $\overset{‐}{\alpha }$is the average of these angles and was used for the calculation of the deflection force $F_{m}$.

Eyelashes from six brands were tested: Arishine®, Lash’d Up™, Pinpoxe®, Lamix, Ardell®, and Lamiya [cf. Fig. 1(a)]. Arishine® and Lamix came with an eyeliner applicator. The material composition of the magnetic eyelashes was not publically available. However, they were all made of permanent magnets, which were most probably neodymium magnets.^3^

Figure 1(b) exhibits the magnetically induced deflection force measurement configuration, while Figure 1(c) depicts the physical coordinate system of the scanner. Initially, we intended to do the test at the location within the scanner where the spatial gradient of the field strength is within 20 percent of its maximum value along z, as described in Ref. [2]. Although the document provided by the manufacturer of the scanner did not have isogradient lines in this region, the spatial gradient forms a plateau centered at about (x, y, z) = (0, 0, 830) mm with values ranging from 3 to 5 T/m. However, when a magnetic eyelash was brought close to this point, it was pulled toward the wall of the bore (i.e. away from the bore axis), leading to a deflection angle that was more than 90 degrees. Therefore, the measurement location was chosen as (x, y, z) = (0, 0, 1078) mm, where the value of the isogradient line was available and reported as 3 T/m. The magnetic field strength at this location was <1 T. Given that common commercial permanent magnets like neodymium magnets do not become saturated at such low magnetic field strengths,^4^ it is reasonable to assume that the deflection force was proportional to the product of the field strength and the spatial field gradient.

In accordance with the ASTM standard,^2^ each piece of magnetic eyelash was deflection‐tested three times. The weights of the strings were also recorded. The magnetically induced deflection force was calculated using the mean deflection angle.

The eyelashes of some brands — Lash’d Up™ (large piece), Pinpoxe®, Ardell®, and Lamiya (both pieces) — showed bending, related to the magnet base consisting of multiple small magnets rather than a single continuous piece. Furthermore, the string length required to knot the string both at the protractor and the magnetic eyelash showed some variation. Consequently, for each eyelash, the location of the protractor was adjusted until the eyelash reached the location of measurement cited above, making the lengths of the strings irrelevant. Due to the tilting of some magnetic eyelashes and in order to prevent the lash‐component of the eyelash from touching the ruler (which was fixed at z = 1078 mm), the eyelashes were kept within a few millimeters of the target location (x, y, z) = (0, 0, 1078) mm. It is worth noting that the spatial gradient values change on a more macro scale (fractions of meters), so a margin of a few millimeters exerts only a negligible effect.

## 3 RESULTS

Figure 1(d) lists the measurement results. The measured deflection angles were similar across different brands.

## 4 DISCUSSION

With the deflection angles being much larger than 45°, the results confirm that ferromagnetic eyelashes are MR unsafe and should be removed before entering Zone III, the space before entering the MRI scanner room.^5^ Furthermore, with the deflection angles being similar across different brands, the heavier magnetic eyelashes are expected to experience a larger projectile effect. Finally, given the effect of the spatial gradient field component of the attraction force, the projectile effect can be significant, even at 1.5 T.

It is worth noting that the weights of the strings were more than 1% of those of the eyelashes, which were extremely light‐weight by design. This exceeds the limit recommended by the ASTM standard and thus requires an explanation.^2^ The excess weight would be a concern if the deflection angles were small since the string weight would be suspected to shadow the true deflection forces. However, in our study, it strengthens the case against magnetic eyelashes because the deflection angles were close to 90° despite this additional load. If it were feasible to use extremely light strings, the deflection angles would be larger.

Failing the deflection test does not make a piece of equipment MR unsafe in and of itself; nevertheless, near the MRI scanner, the eyelashes can rapidly become a moving projectile as close as millimeters from the orbit of the eye and hence become a safety concern. In addition, even if they do not become a direct hazard to the patient, small ferromagnetic objects that get pulled into the MR system can remain there, or lodge within a receiving coil or accessory, potentially resulting in artifacts that may masquerade as pathology. Moreover, they could be relocated to hard‐to‐check areas, and troubleshooting could involve substantial scanner downtime.

The magnetic eyeliner associated with some of these eyelashes are allowed to contain synthetic iron oxide under the current regulations of the FDA, 21 CFR §73.2250. It has been reported that, even with tattooed eyeliners that typically contain iron oxide, adverse events are unlikely, transient, and mild.^6^ However, like the false eyelashes themselves, magnetic eyeliners should be removed prior to the scan in order to ensure safety.

## CONFLICT OF INTEREST

The authors have no conflict of interest to disclose.
